# Design Optimization of SBS-Modified Asphalt Mixture Reinforced with Eco-Friendly Basalt Fiber Based on Response Surface Methodology

**DOI:** 10.3390/ma11081311

**Published:** 2018-07-29

**Authors:** Wensheng Wang, Yongchun Cheng, Guojin Tan

**Affiliations:** College of Transportation, Jilin University, Changchun 130025, China; wangws17@mails.jlu.edu.cn (W.W.); chengyc@jlu.edu.cn (Y.C.)

**Keywords:** asphalt mixture, basalt fiber, response surface methodology, design optimization

## Abstract

This paper investigates the effects of basalt fiber content, length and asphalt-aggregate ratio on the volumetric and strength properties of styrene-butadiene-styrene (SBS)-modified asphalt mixture reinforced with eco-friendly basalt fiber. An experimental scheme was designed to optimize three preparation parameters for the Marshall test indices based on response surface methodology (RSM). The results showed that basalt fiber content presents a more significant effect on air voids, voids in mineral aggregates and voids filled with asphalt. Basalt fiber length is more related to Marshall stability, and flow value exhibits a significant variation trend with asphalt-aggregate ratio. The optimization of preparation parameters is determined as follows: basalt fiber content is 0.34%, length is 6 mm, asphalt-aggregate ratio is 6.57%, which possesses favorable and reliable accuracy compared with experimental results. Furthermore, basalt fiber reinforced asphalt binder and mixture were also studied, and it was found that basalt fiber can enhance the performance of asphalt binder and mixture in terms of cone penetration, softening point, force ductility, as well as pavement performance tests.

## 1. Introduction

Asphalt mixture is a widely used pavement material around the world due to its superior performance. Stone matrix asphalt (SMA) is a gap-graded hot mix asphalt (HMA) mixture that was developed in Germany in the 1960s, and has more coarse aggregates and filler, higher asphalt content, modified asphalt and fibers compared with typical dense-graded asphalt mixtures [1]. Due to the high rutting resistance and durability, SMA has been widely used around the world, and SMA has been almost exclusively used for expressway as surface courses in China since 1992 [2]. As an indispensable part of SMA, fiber can stabilize asphalt binder to improve the performance of SMA. Previous studies have shown that fibers can improve rutting resistance, moisture susceptibility and prevent reflective cracks of asphalt mixtures [3,4,5,6].

Nowadays, various kinds of fibers are commonly applied to asphalt pavement, including lignin fiber, polyester fiber, glass fiber, and so on. As a new eco-friendly mineral fiber, basalt fiber is made of basalt rocks with high mechanical performance, low water absorption and appropriate temperature range [7]. The production of basalt fibers produces less waste and can be degraded directly in the environment without any harm after abandonment. Previously, a lot of research has been carried out to study the influences of basalt fiber on improving the performance of asphalt binder and its mixture by researchers and pavement engineers. Wang et al. [8] investigated the influences of basalt fiber on bitumen binder and mastic at low temperatures by using direct tension and fatigue tests. The results indicated that due to adding basalt fiber, the break stress of binder increased by about 4.5% and the stiffness modulus of mastic increased by 26%. Meanwhile, basalt fiber resulted in improving the fatigue-resistance property of binder and mastic to some extent. Gu et al. [9] analyzed the characteristics of basalt fiber and found that basalt fiber exhibits excellent strength, durability and suitability. Results revealed that the rheological performance of basalt fiber modified asphalt mastic are promoted and remarkable at higher temperature. Cheng et al. [10] indicated that basalt fiber can enhance the high- and low-temperature properties of asphalt mastics in terms of softening point, cone penetration, viscosity, force ductility, DSR and BBR tests. Zhang et al. [11,12] studied the rheological, viscoelastic characteristics of basalt fiber reinforced asphalt mastic and mortar based on 3D model and numerical analysis. Numerical analysis showed that basalt fiber leads to stress redistribution of asphalt mortar and decreases the stress. Moreover, the effects of basalt fiber content and aspect ratio on the shear and compressive properties of matrix were also discussed. Qin et al. [13] carried out an investigation on the properties of basalt fiber modified bitumen mastics with various fiber length and content. Basalt fiber with 6 mm length had much better bitumen adsorption and strength. Compared with lignin fiber and polyester fiber, basalt fiber-modified bitumen mastic presents the best comprehensive properties. In addition, Gao [14] investigated the performance of basalt fiber modified asphalt mixtures and showed that the low-temperature cracking resistance can be significantly improved. Morova [15] used the Marshall stability test to study the application of basalt fiber in asphalt mixture. The optimum asphalt content was determined, and a series of experiments was conducted with different fiber ratios.

However, not much study has been conducted on the design method of basalt fiber-reinforced asphalt mixture, based on the previous literature review. From the previous literature, it is worth noting that fiber content, fiber length and asphalt content play key roles for the performance of fiber-reinforced asphalt mixture [16]. Generally, there are two design methods for asphalt mixtures—the Marshall and Superpave methods—in which the volumetric properties are regarded as responses to design of more reliable asphalt mixtures. Response surface methodology (RSM) is more effectively used for analyzing and optimizing experimental responses, and is becoming more and more popular in the construction field [17]. Tan et al. [18] used the central composite design (CCD) method in conjunction with RSM for the optimal proportion of raw materials in terms of different test indices of asphalt binder. Kavussi et al. [19] investigated and analyzed the effects of preparation parameters including gradation, aggregate, etc., on indirect tensile strength (ITS) of warm mix asphalt (WMA) mixtures based on RSM. Hamzah et al. [20,21] investigated the influences of recycled aggregate content, compaction temperature and asphalt content on volumetric and mechanical properties of asphalt mixture for optimizing the binder content based on the CCD of RSM. Furthermore, Hamzah et al. [22] found that statistical models based on RSM can predict the effects of aging on binder viscosity behavior at high temperatures effectively. Khodaii et al. [23] employed RSM to evaluate the effects of lime content and gradation on ITS and its ratio of asphalt mixture under dry and saturated conditions. Haghshenas et al. [24] utilized RSM for optimizing the bitumen content and gradation of HMA mixtures based on tensile strength ratio. Based on the extensive literature, RSM can be successfully applied to study asphalt binder and mixture.

In this study, an experimental scheme was designed for styrene-butadiene-styrene (SBS)-modified asphalt mixture reinforced with eco-friendly basalt fiber based on RSM. The relationships between preparation parameters and Marshall test indices including volumetric and strength properties were analyzed to evaluate the effects of basalt fiber content, length and asphalt-aggregate ratio. A design optimization of basalt fiber and SBS-modified asphalt mixture was proposed and validated with experimental results. Meanwhile, the performance of basalt fiber-modified asphalt binder and mixture was also studied.

## 2. Materials and Methods

### 2.1. Raw Materials

**Figure 1:**
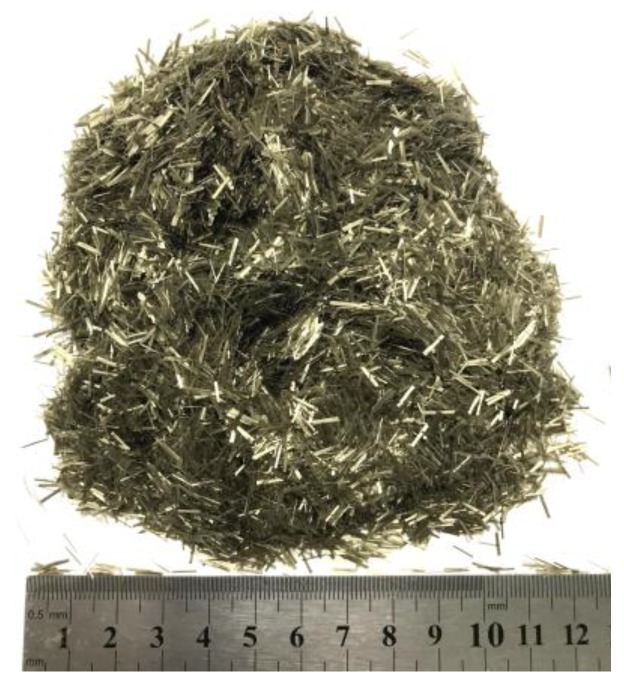
Morphology of basalt fiber with length of 6 mm in this study.

In this study, SBS-modified asphalt was chosen, and its basic physical properties are listed in Table 1. The coarse and fine aggregates, as well as fillers, were obtained from a local quarry in Yitong of Jilin Province, China. Their physical properties are shown in Table 2. Basalt fiber with different lengths was used to modify the asphalt binder and mixture, as shown in Figure 1, and its basic properties are summarized in Table 3.

### 2.2. Sample Preparation

Basalt fibers with different lengths of 3, 6 and 9 mm were used in this study. According to the previous literature [9,10,15,25], the percentage of basalt fiber to asphalt binder should not exceed 5%. Therefore, in order to investigate the influence of basalt fiber at the level of asphalt binder, their proportions added into SBS-modified asphalt were 0%, 1%, 2%, 2.5%, 3%, 3.5% and 4% by mass of SBS-modified asphalt, respectively. Based on previous research [10,14,26], the detailed preparation procedures of SBS-modified asphalt binder reinforced with basalt fiber are as follows: (i) basalt fiber with different lengths and SBS-modified asphalt were heated in an oven at 170 °C until the constant weight and stable state; (ii) three kinds of basalt fiber with different lengths were added into SBS-modified asphalt at seven different proportions, and twenty-one experimental groups could be obtained; (iii) in order to ensure that basalt fibers can be distributed uniformly in asphalt binder, the mixture of basalt fiber and asphalt was placed in a shear homogenizer (KRH-I, Shanghai Konmix Mechanical & Electrical Equipment Technology Co. Ltd., Shanghai, China) after a preliminary manual blending. Then, shearing temperature and speed were set as 170 °C and 6000 rev/min, respectively. After mixing of asphalt with basalt fibers for one hour, SBS-modified asphalt containing basalt fiber was prepared.

**Figure 2:**
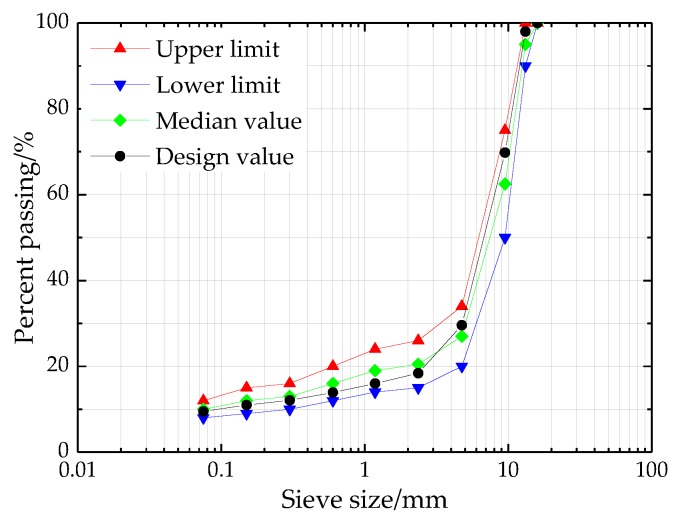
Gradation of stone matrix asphalt (SMA)-13 used in this study.

Asphalt mixture specimens were produced to investigate the design optimization of SBS-modified asphalt mixture reinforced with basalt fiber. Figure 2 illustrates the gradation of SMA with a nominal maximum size of 13.2 mm. According to the Chinese specification JTG E20-2011 [27], the Marshall specimens of asphalt mixture with height of 63.5 mm and diameter of 101 mm were made by Marshall procedures, which were used for laboratory tests and optimization analysis based on RSM. The detailed preparation procedures of SBS-modified asphalt mixture reinforced with basalt fiber are as follows: (i) the aggregates and fillers were weighted and placed in an oven at 180 °C for two hours and SBS-modified asphalt was heated to 170 °C; (ii) the weighted aggregates and basalt fiber were blended together in a mixing pot and then asphalt was poured and mixed at 165 °C until the aggregates were coated; (iii) the weighted fillers were added and mixed well at 165 °C; (iv) asphalt mixtures were compacted with 50 blows of Marshall hammer per side for the target of 4% air void content.

### 2.3. Testing Procedure

#### 2.3.1. Cone Penetration Test

**Figure 3:**
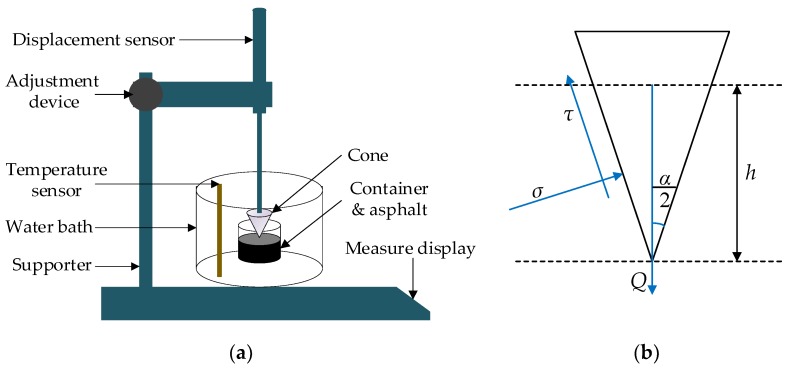
Cone penetration test [28]: (**a**) diagram of cone penetration test; (**b**) cone structure.

Cone penetration test, as shown in Figure 3, is a method to characterize the shearing resistance of fiber modified asphalt, which was developed by Chen [28]. In the cone penetration test, basalt fiber-modified asphalt binder samples were prepared after melting and cooling under controlled conditions. Afterwards, a cone of penetration instrument penetrated into an asphalt binder sample from the sample surface and the sink depth in a stable state can be measured and obtained. It should be noted that penetration depth would be smaller than the sample height. By using the equilibrium equation of force, the shear stress (*τ*) of SBS-modified asphalt reinforced with basalt fiber could be calculated as follows:

*τ* = [981*Q*cos^2^(*α*/2)]/[π*h*^2^tan(*α*/2)], (1)

where *Q* is the cone mass (150 g), *h* is the sink depth (0.1 mm), *α* is the cone angle (30°). Three replicate samples were used for each experimental group of modified asphalt binders.

#### 2.3.2. Softening Point Test

Softening point of asphalt is defined as the temperature at which asphalt can’t bear a steel ball weighing 3.5 g. Therefore, the softening point test is the basic method for the high-temperature susceptibility evaluation of asphalt. According to JTG E20-2011 [27], two replicate samples were prepared and measured for each group of SBS-modified asphalt reinforced with basalt fiber by using the ring and ball apparatus.

#### 2.3.3. Force Ductility Test

The force ductility test refers to the force measurement during the elongation of asphalt at low temperatures, which was firstly introduced by Anderson and Wiley since 1976 [29]. Previous research has shown that the force ductility test is an effective modified ductility method to indicate the low-temperature performance and tensile property of asphalt [30]. In this study, three replicate asphalt samples were prepared in ductility molds in a water bath of 5 °C and then force ductility testing was carried out at a deformation rate of 50 mm/min. Based on the recorded force-elongation curve, strain energy, also called deformation energy, can be calculated by measuring the area under the recorded curve between 20 and 40 mm.

#### 2.3.4. Marshall Test Method

SBS-modified asphalt mixture specimens containing basalt fiber were prepared using the Marshall test method in this study, which is a very popular design method of asphalt mixture due to the relative simplicity, economical equipment and procedure. Then, different parameters of compacted asphalt specimens can be measured, including Marshall stability (*MS*), flow value (*FV*), air voids (*VA*) and voids in mineral aggregates (*VMA*), as well as voids filled with asphalt (*VFA*). Before testing, specimens were immersed in water at 60 °C for 0.5 h. Then, following the Chinese specification [27], a constant compressive loading was applied on cylindrical asphalt specimens at a speed of 50 mm/min until failure occurs. The maximum loading and deformation are defined as *MS* and *FV*, respectively. *VA*, *VMA* and *VFA* can be obtained by the given calculation equations below:

*VA* = [1 − *γ_f_*/*γ_TMD_*] × 100, (2)

*VMA* = [1 − *γ_f_* × *P_s_*/*γ_sb_*] × 100*,* (3)

*VFA* = [(*VMA* − *VA*)/*VMA*] × 100*,* (4)

where *γ_f_* is the bulk specific gravity, *γ_TMD_* is the theoretical maximum specific density, *P_s_* is the aggregate content percent by weight of mixture, *γ_sb_* is the bulk specific gravity of aggregates.

#### 2.3.5. Pavement Performance Test

Based on the optimal modified asphalt mixture design, pavement performance testing was carried out to evaluate the engineering properties of the optimal modified asphalt mixture in this study, including the high-temperature rutting resistance by wheel tracking test [27], the low-temperature cracking resistance by indirect tensile stiffness modulus (ITSM) test [6] and the moisture stability by immersion Marshall and freeze-thaw splitting tests [27]. The detailed experimental processes of the wheel tracking test, immersion Marshall and freeze-thaw splitting tests have been described in previous studies [31,32]. The ITSM test was conducted to characterize the low-temperature properties of asphalt mixture, which is a widely used experimental method of measuring tensile properties. In this study, the Marshall specimens were prepared and then put in a chamber for at least 6 h at 0 °C before tested. Then a servo-pneumatic universal testing machine (NU-14, Cooper Research Technology, Ltd., Ripley, UK) was employed for ITSM test at a loading speed of 1 mm/min. The target horizontal deformation was set as 5 μm, and the peak value of loading could be obtained at the target deformation. The stiffness modulus can be calculated by using the following equation:

*S_m_* = [*F* × (*μ* + 0.27)] / (*h* × *Z*)*,* (5)

where *S_m_* is the stiffness modulus, *F* is the peak load, *μ* is the Poisson ratio, *h* is the specimen height, *Z* is the measured horizontal deformation.

### 2.4. Response Surface Methodology

**Figure 4:**
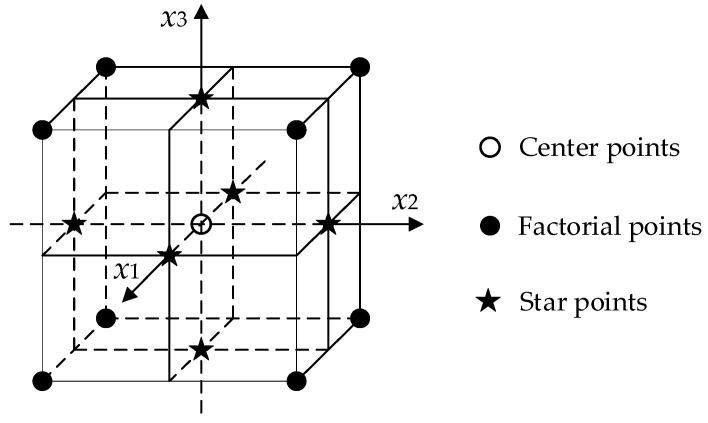
A three-factor layout for face-centered central composite design (FCCD).

RSM is a statistical method for optimizing random experimental processes, which is used to explore the quantitative relationship between independent variables and response variables [17]. A response surface model is established, and a suitable fitting model based on test data can be chosen to determine the optimum experimental conditions and procedure. In general, CCD is a common experimental design method in RSM, which is a fractional factorial experiment design. Then, the face-centered central composite design (FCCD) was adopted to investigate the relationship between independent variables and response variables for determining a suitable experimental formulation [33]. A three-factor layout for the FCCD is shown in Figure 4, which is a cube with axial points on the face centers. The number of experimental samples can be given by (2*^k^* + 2*k* + *n*), in which *k* represents the number of factors, *n* is the number of center points.

The experimental design in this study required nineteen experimental runs, which was composed of eight factorial points, five center points and six star points at three experimental levels (*k* = 3, *n* = 5). Center points were set as five because some replication should be required to estimate the experimental error. These three independent variable factors are basalt fiber content (*X*_1_), basalt fiber length (*X*_2_) and asphalt-aggregate ratio (*X*_3_), which are abbreviated as *BFC*, *BFL* and *AAR*, respectively. The quantitative relationship between the amount of added basalt fibers in asphalt binders and mixtures is not clear, but the approximate percentage range of basalt fiber to asphalt is basically consistent in asphalt binder and mixture. From the previous literature [15,16,25,34], the appropriate basalt fiber content should exceed 0.5%. Additionally, based on the previous research [15,35,36,37,38], the optimum asphalt content of modified SMA mixtures can reach up to approximately 6.7%. In view of the added basalt fibers in this study varying from 3 to 9 mm, the basalt fiber content and asphalt-aggregate ratio were chosen for appropriate ranges accordingly. Table 4 lists three independent factors at three experimental levels, in which coded levels are the normalized levels and “−1, 0 and +1” stand for the low, medium and high levels of independent factors, respectively. Thus, the experimental processes can be optimized for *MS* (*Y*_1_), *FV* (*Y*_2_), *VA* (*Y*_3_), *VMA* (*Y*_4_) and *VFA* (*Y*_5_).

The Design-Expert 8.0 software (Stat-Ease, Inc., Minneapolis, MN, US) was employed for the experimental design, response surface modeling, statistical regression analysis and process optimization. Based on the experimental data, it is well suited for fitting a quadratic surface model using the following equation:

(6) $$y=\beta_{0}+\sum_{i=1}^{k}\beta_{i}x_{i}+\sum_{i=1}^{k}\beta_{ii}x_{i}^{2}+\sum_{i<j}^{k}\beta_{ij}x_{i}x_{j}+\varepsilon ,$$

where *y* is the response, *x_i_* and *x_j_* are the coded independent variables, *β*_0_ is the mean value of response constant coefficient, *β_i_* is the linear effect of independent variable *x_i_*, *β_ii_* is the secondary effects of *x_i_*, *β_ij_* is the linear interaction between *x_i_* and *x_j_*, *ε* is the random error.

## 3. Results and Discussion

### 3.1. Test Results of SBS-Modified Asphalt Binder Reinforced with Basalt Fiber

**Figure 5:**
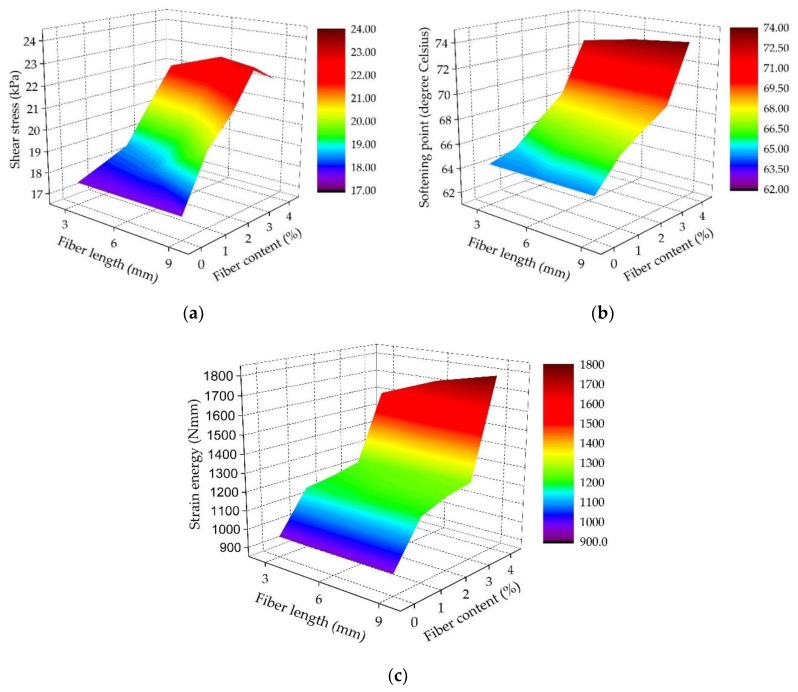
Test results of modified asphalt binders in this study: (**a**) Cone penetration test; (**b**) Softening point test; (**c**) Force ductility test.

Figure 5 shows the cone penetration test result at 30 °C, the softening point result and the strain energy result of SBS-modified asphalt binder reinforced with basalt fiber. It can be clearly seen that these test results for modified bitumen binder varied with the fiber length and content. Furthermore, the fiber length had a slight influence on the softening point and strain energy results of bitumen binder when the basalt fiber content was lower. With the increment of basalt fiber content, the changes of modified asphalt binder became more and more significant.

As seen in Figure 5a, basalt fiber can improve the shear resistance of asphalt binder compared to the asphalt binder without fiber. With the addition of basalt fiber, the shear strength of modified asphalt binder gradually increased. This is because basalt fiber can lead to a spatial networking structure in the asphalt binder. On the other hand, fibers can also absorb the light components in asphalt, which saturates it and increases its viscosity [28,38]. Thus, the addition of basalt fiber resulted in a smaller cone penetration and a higher shear strength. However, it should be noted that the shear resistance of modified asphalt binder decreases under the conditions of higher basalt fiber content and larger fiber length, which could be attributed to the uneven distribution of basalt fiber in the asphalt binder. Consequently, it is believed that suitable basalt fiber content and fiber length should be crucial for asphalt properties and its production process.

Figure 5b illustrates that the softening point results for modified asphalt binders increased with increasing fiber content and length. Generally, the softening point is used to evaluate the temperature susceptibility of asphalt materials, and a higher softening point means a lower temperature susceptibility. As a result, it is evident that basalt fiber can well improve the high-temperature susceptibility of asphalt binder. This is because the addition of basalt fiber into asphalt leads to less bitumen, higher stiffness, and bitumen absorption, resulting, to a certain extent, in a reinforcement mechanism, filler-like action, and increased viscosity.

Asphalt material is a typical viscoelastic material, and asphalt exhibits elastic characteristics at low temperatures. The work done by the external force on asphalt at low temperatures is stored as elastic strain and is converted into surface energy when fracture occurs. Normally, the higher the strain energy is, the better the tensile properties are [30]. As demonstrated in Figure 5c, the strain energy results of the modified asphalt binders clearly increased with the increase of basalt fiber content and length. It is worth noting that the variation trend of strain energy was especially significant when the basalt fiber content exceeded 3%. This indicates that adding more basalt fiber could greatly improve the low-temperature tensile properties of asphalt binder.

### 3.2. Analysis and Optimization of SBS-Modified Asphalt Mixture Reinforced with Basalt Fiber Using RSM

#### 3.2.1. Experimental Design and Test Results Based on FCCD

RSM was adopted to investigate the influences of basalt fiber content, fiber length and asphalt-aggregate ratio on the Marshall test indices of modified asphalt mixtures. By using FCCD, an experimental design was established with 19 experimental groups, and the total number of experimental groups could be greatly reduced. Table 5 details the experimental design and Marshall test results by using the FCCD. Preparation parameters including *BFC*, *BFL* and *AAR* were considered to be independent variables, and *MS*, *FV*, *VA*, *VMA* and *VFA* were responses or dependent variables.

#### 3.2.2. Statistical Analysis and Discussion

##### Analysis of Variance (ANOVA) Results

According to the above experimental design and test outcomes using FCCD, the superior regression model was suggested and chosen for the further analysis. The superior model was determined based on *R*-squared, Adjusted *R*-squared (Adj. *R*-squared), Adeq. precision, Fisher’s test value (*F*-value) and the probability “Prob > *F*-value” (*p*-value) among different responses, including linear, two-factor interaction (2FI), quadratic, and cubic polynomials. The analysis of variance (ANOVA) was adopted to evaluate the statistical significance of independent variables (i.e., preparation parameters) and their interactions on the responses. The statistical significance level was chosen as 0.05; that is, models and factors can be considered significant when the *p*-value falls below 0.05. The Design-Expert 8.0 software indicated that the quadratic model looks best, and these terms are significant. Corresponding ANOVA results for quadratic models and independent variables were obtained and are listed in Table 6 and Table 7, respectively.

##### Analysis of Marshall Stability (*MS*)

The ANOVA results of *MS* (*Y*_1_) are listed in the first row of Table 6. The results illustrate that the quadratic model of *MS* possessed satisfactory fitting levels, in which *R*-squared is 0.9834, Adj. *R*-squared is 0.9668, and they are close to one. Additionally, Adeq. precision stands for the signal-to-noise ratio, and it is desirable when this ratio exceeds 4. The Adeq. precision of *MS* is 21.472, and thus greater than 4, which indicates an adequate signal, meaning that this model can be used to navigate the design space.

Based on the ANOVA results for independent variables in Table 7, the factors of the quadratic model of *MS* were demonstrated in detail. The *p*-values quantify the significance of these factors in the quadratic model of *MS*, in which the significance level was chosen as 0.05. A factor with *p*-value less than 0.05 means a statistically significant relationship between this factor and *MS*. Therefore, the significant factors in the quadratic model of *MS* include *X*_1_, *X*_2_, *X*_3_, (*X*_1_)^2^, (*X*_2_)^2^ and (*X*_3_)^2^. Based on the least squares method, the regression coefficients of factors can be determined. Then, by leaving out the insignificant factors, the reasonable second-order polynomial equation in terms of actual factors for *MS* can be established as:

*Y*_1_ = −1099.95 + 15.97*X*_1_ + 2.94*X*_2_ + 334.19*X*_3_ − 18.63(*X*_1_)^2^ − 0.17(*X*_2_)^2^ − 25.36(*X*_3_)^2^*,* (7)

**Figure 6:**
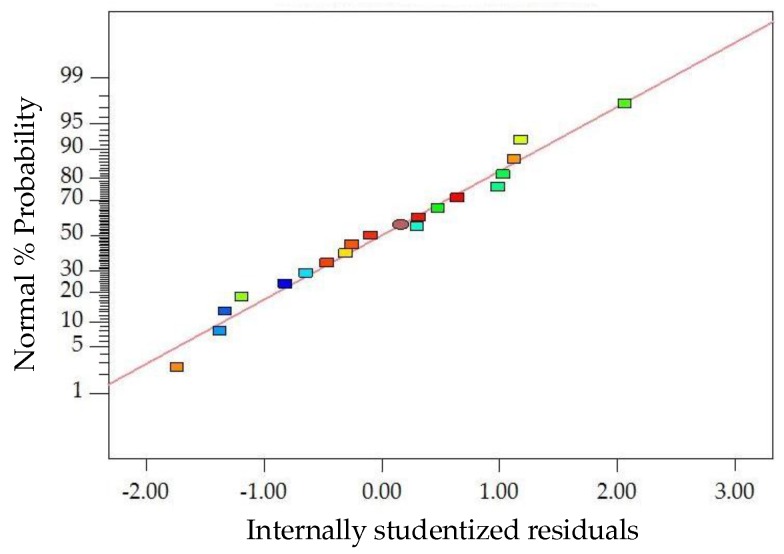
Diagnostics of statistical model: normal plot of residuals for *MS*.

**Figure 7:**
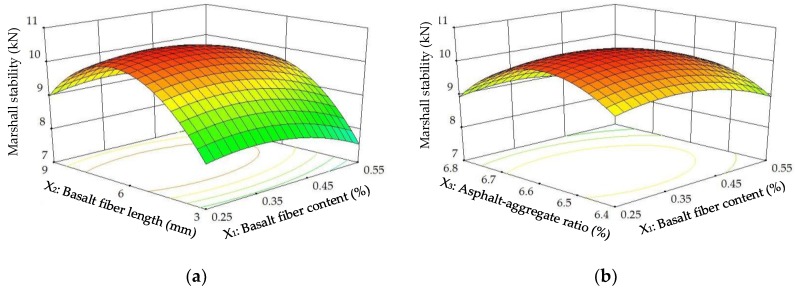

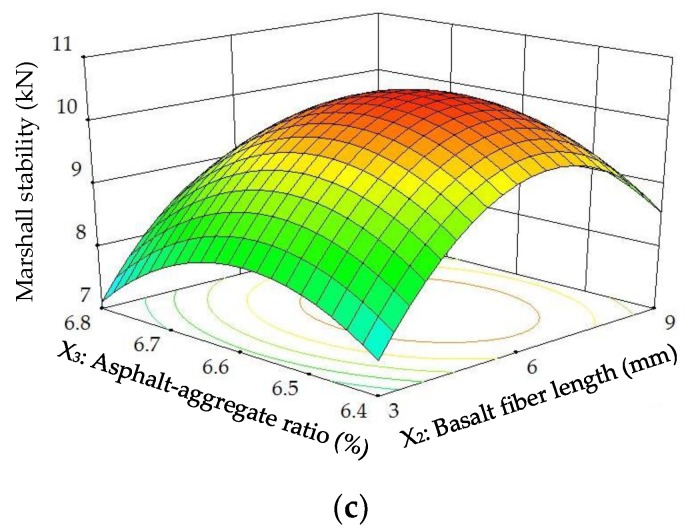
Response surface plots between *MS* and factors: (**a**) Factors: *BFC* and *BFL* at *AAR* = 6.6%; (**b**) Factors: *BFC* and *AAR* at *BFL* = 6 mm; (**c**) Factors: *BFL* and *AAR* at *BFC* = 0.4%.

The diagnostics of the statistical model in Figure 6 presents an approximately linear set of data points, indicating a higher significance. The ANOVA results indicate that the linear terms and quadratic terms of basalt fiber content, fiber length and asphalt-aggregate ratio are significant model terms, in which the quadratic term of basalt fiber length has the most significant effect on *MS* of modified asphalt mixture, with *p*-value < 0.0001. Subsequently, Figure 7 illustrates the three-dimensional (3D) response surface and two-dimensional (2D) contour plots for *MS*, which are plotted by the fitting quadratic polynomial equation to reveal the relationship between preparation parameters and *MS*, as well as the interaction of preparation parameters.

As illustrated in Figure 7, the *MS*s of modified asphalt mixture firstly presented increasing trends, and then decreased when the basalt fiber content or fiber length or asphalt-aggregate ratio increased continuously. These results could be attributed to a spatial networking structure and fiber’s adhesion in asphalt mixture by the addition of basalt fiber, in which fiber has a reinforcement effect on asphalt material for a networking structure. However, *MS*s decreased at higher basalt fiber content and larger fiber length, which is because it would be difficult for basalt fibers to disperse uniformly in asphalt, and more asphalt is needed to wrap around the surfaces of fiber and aggregates to form effective interface adhesions. In other words, due to the higher basalt fiber content and larger fiber length, fibers in asphalt easily coagulated together, resulting in weak points. Meanwhile, a lower asphalt-aggregate ratio led to an SMA structure without sufficient filling asphalt, meaning that the structure became looser, while too much asphalt may cause it not to form a dense interlocking structure.

##### Analysis of Flow Value (*FV*)

The ANOVA results of *FV* (*Y*_2_) are listed in the second row of Table 6. *R*-squared is 0.9796, Adj. *R*-squared is 0.9591 and Adeq. precision of *FV* is 25.279, illustrating that the quadratic model of *FV* also possessed satisfactory fitting levels. As listed in Table 7, the factors of the quadratic model of *FV* have been demonstrated in detail. Based on the *p*-values, the significant factors in the quadratic model of *FV* were obtained, i.e., *X*_1_, *X*_2_, *X*_3_, *X*_1_*X*_3_ and (*X*_3_)^2^. Based on the least squares method, the regression coefficients of factors can be determined, and the reasonable second-order polynomial equation in terms of actual factors for *FV* can be established as:

*Y*_2_ = 415.99 + 44.40*X*_1_ + 0.27*X*_2_ − 130.49*X*_3_ − 6.30*X*_1_*X*_3_ + 10.29(*X*_3_)^2^*,* (8)

**Figure 8:**
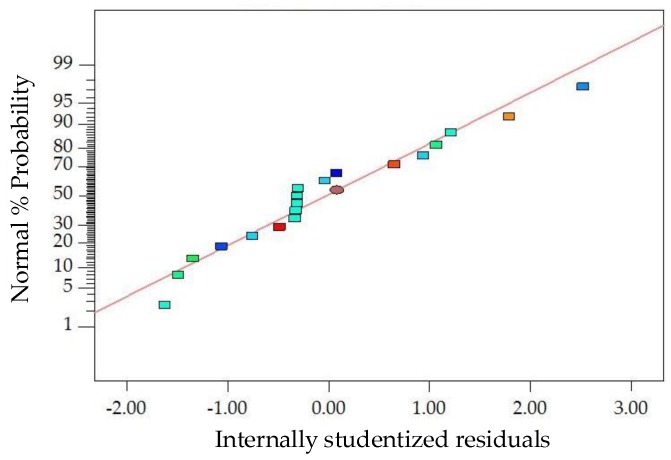
Diagnostics of statistical model: normal plot of residuals for *FV*.

**Figure 9:**
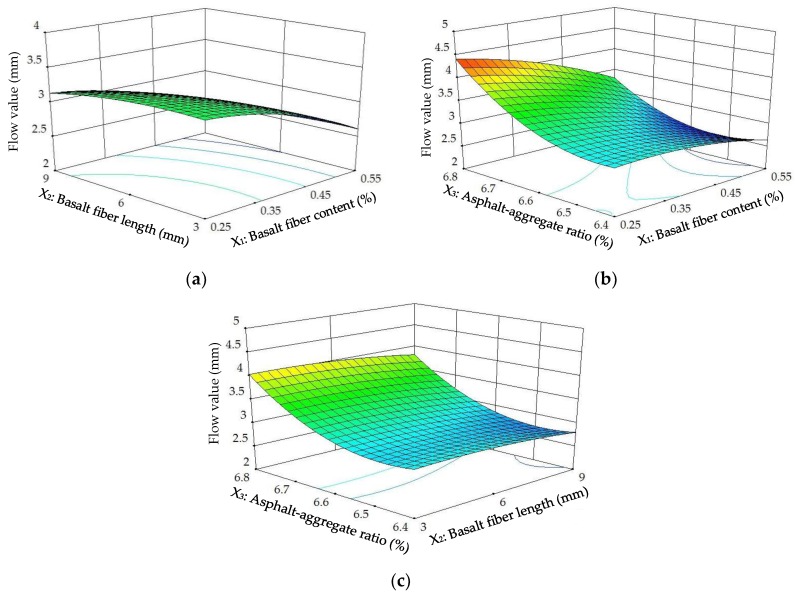
Response surface plots between *FV* and factors: (**a**) Factors: *BFC* and *BFL* at *AAR* = 6.6%; (**b**) Factors: *BFC* and *AAR* at *BFL* = 6 mm; (**c**) Factors: *BFL* and *AAR* at *BFC* = 0.4%

The diagnostics of the statistical model in Figure 8 presents an approximately linear set of data points, indicating a higher significance. The ANOVA results indicate that the linear terms of basalt fiber content, fiber length and asphalt-aggregate ratio, the quadratic term of the asphalt-aggregate ratio and the interaction terms between basalt fiber content and asphalt-aggregate ratio are significant model terms, in which the quadratic terms of basalt fiber length and asphalt-aggregate ratio have the most significant effects on the *FV* of modified asphalt mixture, with *p*-value < 0.0001. Subsequently, Figure 9 illustrates the 3D response surface and 2D contour plots for *FV*, which are plotted by fitting the quadratic polynomial equation to reveal the relationship between preparation parameters and *FV*, as well as the interaction of preparation parameters.

As demonstrated in Figure 9, the *FV*s of the modified asphalt mixture significantly increased with increasing asphalt-aggregate ratio. Generally, *FV* depends on asphalt content, and asphalt mixtures with higher asphalt content usually have a larger *FV* [16]. Accordingly, *FV* presented a significant variation trend with asphalt-aggregate ratio. On the other hand, *FV*s remained approximately unchanged or slightly decreased with increasing basalt fiber content and length. These trends are consistent with the previous study [5]. Based on the comparative analysis in Figure 9, as well as the ANOVA results, it is evident that the asphalt-aggregate ratio has the most significant effect on *FV*.

##### Analysis of Air Voids (*VA*)

The ANOVA results of *VA* (*Y*_3_) are listed in the third row of Table 6. *R*-squared is 0.9966, Adj. *R*-squared is 0.9932 and Adeq. precision of *VA* is 59.932, illustrating that the quadratic model of *VA* also possessed satisfactory fitting levels. As seen in Table 7, the factors of the quadratic model of *VA* were demonstrated in detail. Based on the *p*-values, the significant factors in the quadratic model of *VA* were obtained, i.e., *X*_1_, *X*_2_, *X*_3_, *X*_1_*X*_3_, (*X*_1_)^2^ and (*X*_3_)^2^. Based on the least squares method, the regression coefficients of factors can be determined, and the reasonable second-order polynomial equation in terms of actual factors for *VA* can be established as:

*Y*_3_ = 830.74 + 22.19*X*_1_ + 0.03*X*_2_ − 255.12*X*_3_ − 14.61(*X*_1_)^2^ + 19.53(*X*_3_)^2^*,* (9)

**Figure 10:**
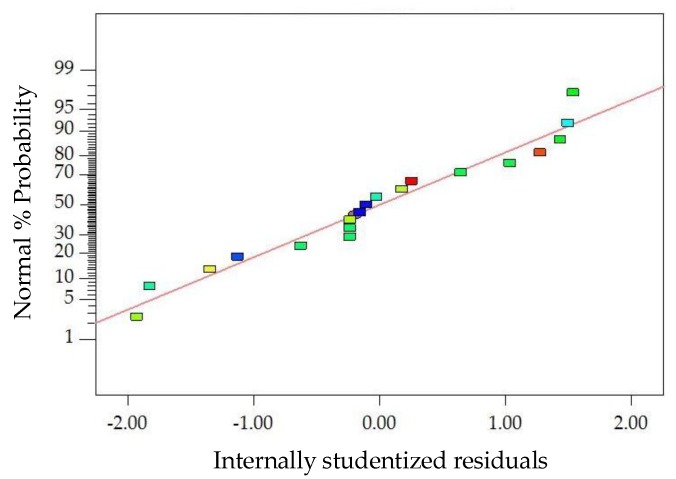
Diagnostics of statistical model: normal plot of residuals for *VA*.

**Figure 11:**
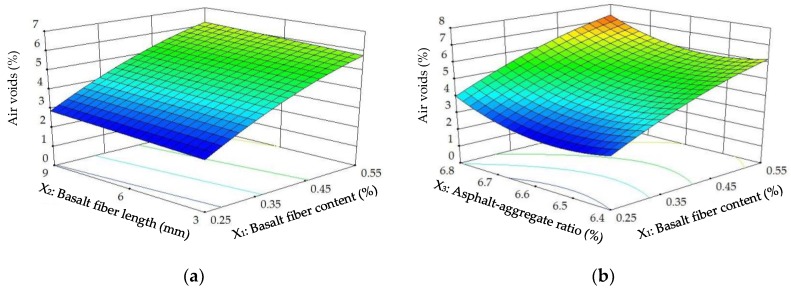

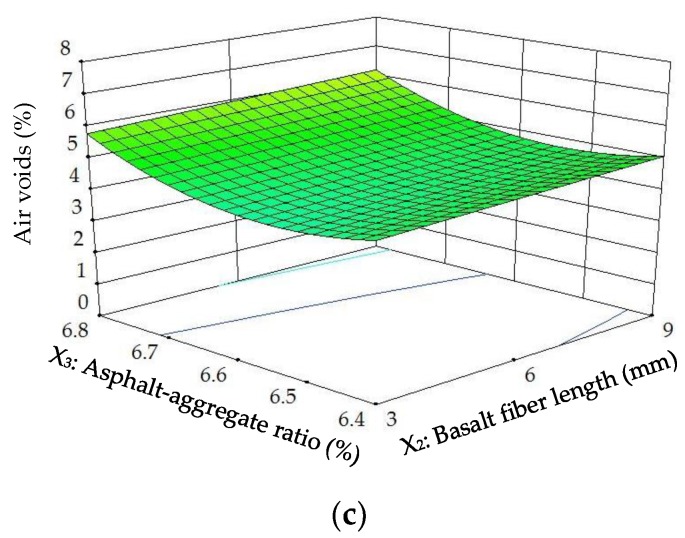
Response surface plots between *VA* and factors: (**a**) Factors: *BFC* and *BFL* at *AAR* = 6.6%; (**b**) Factors: *BFC* and *AAR* at *BFL* = 6 mm; (**c**) Factors: *BFL* and *AAR* at *BFC* = 0.4%.

The diagnostics of the statistical model in Figure 10 present an approximately linear set of data points, indicating a higher significance. The ANOVA results indicate that the linear terms of basalt fiber content, fiber length and asphalt-aggregate ratio and the quadratic terms of basalt fiber content and asphalt-aggregate ratio are significant model terms, of which the linear terms of basalt fiber content and asphalt-aggregate ratio, as well as the quadratic term of asphalt-aggregate ratio, have the most significant effects on the *VA* of modified asphalt mixture, with *p*-value < 0.0001. Subsequently, Figure 11 illustrates the 3D response surface and 2D contour plots for *VA*, which are plotted by fitting the quadratic polynomial equation to reveal the relationship between preparation parameters and *VA*, as well as the interaction of preparation parameters.

As shown in Figure 11, the *VA*s of the modified asphalt mixture exhibited a significant increasing trend with increasing basalt fiber content, and *VA*s remained approximately unchanged at different basalt fiber lengths. With respect to asphalt-aggregate ratio, *VA*s firstly slightly decreased and then increased gradually with increasing ratio. It is evident from the comparative analysis that basalt fiber content and asphalt-aggregate ratio have more significant effects on *VA* than basalt fiber length. Compared with mineral aggregates, basalt fiber has the lowest specific gravity (i.e., 2.55~2.65 < 2.738 as shown in Table 2 and Table 3), which could cause the bulk specific gravity (*γ_f_*) of modified asphalt mixture to decrease after adding basalt fiber. Then, combined with Equation (2), these findings can explain the higher *VA* at higher basalt fiber contents. In addition, asphalt mixtures with optimum asphalt content have much lower *VA*s, as discussed previously [16,31]. Hence, these trends in Figure 11 are consistent with the previous research, and the optimum asphalt content of modified asphalt mixture is between 6.4% and 6.8%.

##### Analysis of Voids in Mineral Aggregates (*VMA*)

The ANOVA results of *VMA* (*Y*_4_) are listed in the fourth row of Table 6. *R*-squared is 0.9968, Adj. *R*-squared is 0.9935 and Adeq. precision of *VMA* is 61.691, illustrating that the quadratic model of *VMA* also possessed satisfactory fitting levels. As shown in Table 7, the factors of the quadratic model of *VMA* were demonstrated in detail. Based on the *p*-values, the significant factors in the quadratic model of *VMA* were *X*_1_, *X*_2_, *X*_3_, *X*_1_*X*_3_, (*X*_1_)^2^ and (*X*_3_)^2^. Then, the regression coefficients of factors can be determined by the least squares method, and the reasonable second-order polynomial equation in terms of actual factors for *VMA* can be established as:

*Y*_4_ = 746.89 + 18.71*X*_1_ + 0.03*X*_2_ − 224.67*X*_3_ − 12.57(*X*_1_)^2^ + 17.18(*X*_3_)^2^*,* (10)

**Figure 12:**
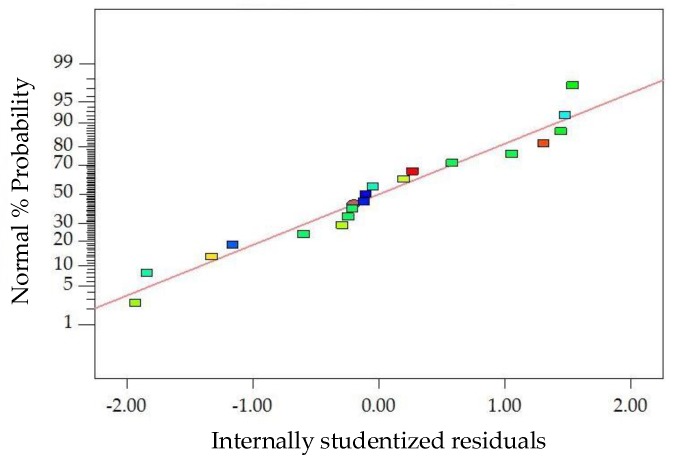
Diagnostics of statistical model: normal plot of residuals for *VMA*.

**Figure 13:**
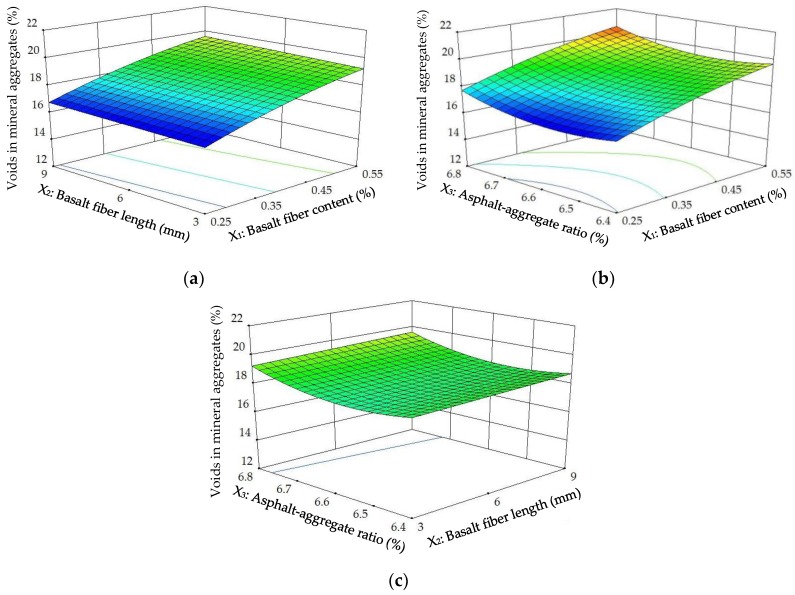
Response surface plots between *VMA* and factors: (a) Factors: *BFC* and *BFL* at *AAR* = 6.6%; (**b**) Factors: *BFC* and *AAR* at *BFL* = 6 mm; (**c**) Factors: *BFL* and *AAR* at *BFC* = 0.4%.

The diagnostics of the statistical model in Figure 12 presents an approximately linear set of data points, indicating a higher significance. The ANOVA results indicate that the linear terms of basalt fiber content, fiber length and asphalt-aggregate ratio and the quadratic terms of basalt fiber content and asphalt-aggregate ratio are significant model terms, in which the linear terms of basalt fiber content and asphalt-aggregate ratio, as well as the quadratic term of asphalt-aggregate ratio, have the most significant effects on the *VMA* of modified asphalt mixture, with *p*-value < 0.0001. Subsequently, Figure 13 illustrates the 3D response surface and 2D contour plots for *VMA*, which are plotted by fitting the quadratic polynomial equation to reveal the relationship between preparation parameters and *VMA*, as well as the interaction of preparation parameters.

As shown in Figure 13, the *VMA*s of modified asphalt mixture exhibited approximately similar variation trends to *VA*. This is expected, due to the decrease of bulk specific gravity (*γ_f_*) of the modified asphalt mixture after adding basalt fiber, as discussed above. The results can also be explained by Equation (3). As a result, the bulk specific gravity influenced by basalt fiber content can be considered a significant factor on *VMA*.

##### Analysis of Voids Filled with Asphalt (*VFA*)

The ANOVA results of *VFA* (*Y*_5_) are listed in the fifth row of Table 6. *R*-squared is 0.9953, Adj. *R*-squared is 0.9905 and Adeq. precision of *VFA* is 50.560, illustrating that the quadratic model of *VFA* also possessed satisfactory fitting levels. As listed in Table 7, the factors of the quadratic model of *VFA* were demonstrated in detail. Based on the *p*-values, the significant factors in the quadratic model of *VFA* included *X*_1_, *X*_2_, *X*_3_, *X*_1_*X*_3_, (*X*_1_)^2^ and (*X*_3_)^2^. Then, the regression coefficients of factors can be determined by the least squares method, and the reasonable second-order polynomial equation in terms of actual factors for *VFA* can be established as:

*Y*_5_ = −3268.68 − 189.14*X*_1_ − 0.58*X*_2_ + 1039.50*X*_3_ + 85.15(*X*_1_)^2^ − 79.98(*X*_3_)^2^*,* (11)

**Figure 14:**
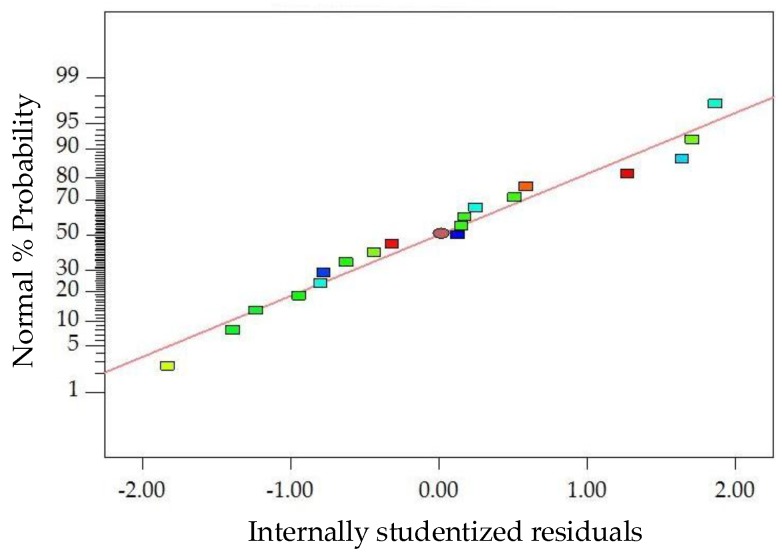
Diagnostics of statistical model: normal plot of residuals for *VFA*.

**Figure 15:**
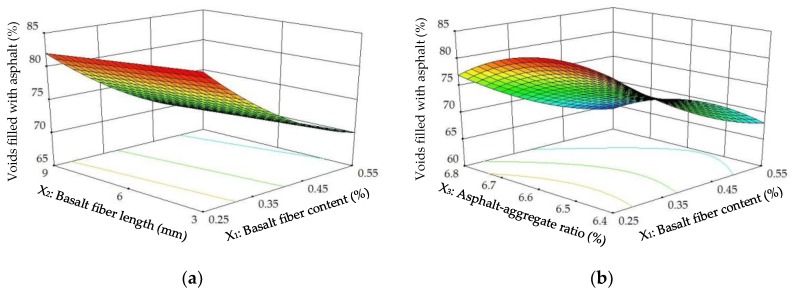

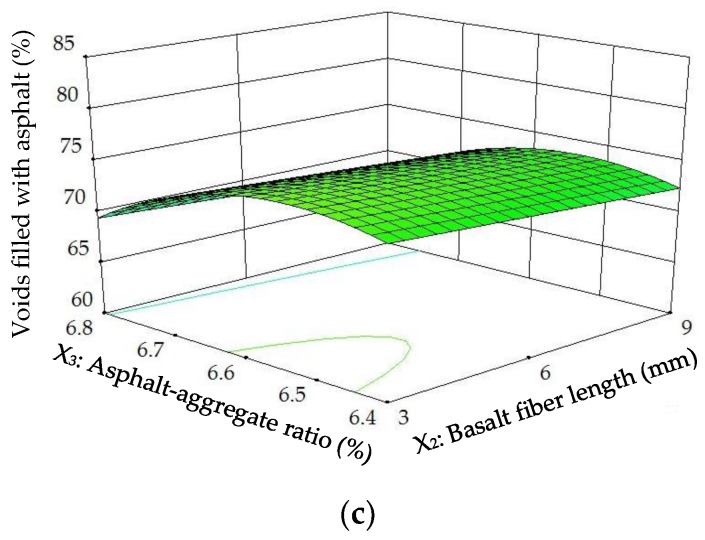
Response surface plots between *VFA* and factors: (**a**) Factors: *BFC* and *BFL* at *AAR* = 6.6%; (**b**) Factors: *BFC* and *AAR* at *BFL* = 6 mm; (**c**) Factors: *BFL* and *AAR* at *BFC* = 0.4%.

The diagnostics of the statistical model in Figure 14 presents an approximately linear set of data points, indicating a higher significance. The ANOVA results indicate that the linear terms of basalt fiber content, fiber length and asphalt-aggregate ratio and the quadratic terms of basalt fiber content and asphalt-aggregate ratio are significant model terms, in which the linear terms of basalt fiber content and asphalt-aggregate ratio, as well as the quadratic term of asphalt-aggregate ratio, have the most significant effects on the *VFA* of modified asphalt mixture, with *p*-value < 0.0001. Subsequently, Figure 15 illustrates the 3D response surface and 2D contour plots for *VFA*, which are plotted by fitting the quadratic polynomial equation to reveal the relationship between preparation parameters and *VFA*, as well as the interaction of preparation parameters.

As illustrated in Figure 15, the *VFA*s of modified asphalt mixture remained approximately unchanged or slightly decreased with increasing basalt fiber length and asphalt-aggregate ratio, whereas *VFA*s significantly decreased with increasing basalt fiber content. This is evidence that basalt fiber content has the most significant effect on *VFA*. Additionally, a lower *VFA* indicates a thinner asphalt film between aggregates, resulting in an unstable interface adhesion. Therefore, higher basalt fiber content is not recommended for avoiding a much lower *VFA*.

#### 3.2.3. Optimization of Preparation Parameters and Model Verification

As discussed above, the preparation parameters exhibit various effects on the response variables of modified asphalt mixture, and the corresponding influence significance levels are also different from each other. In order to determine the optimal combination of preparation parameters, a multiple response optimization was carried out based on the best fitted response surface model. The target values were selected in accordance with JTG F40-2004 [39], which are presented in Table 8. Then, the optimal combination of preparation parameters can be obtained based on RSM by the Design-Expert 8.0 software, and Table 9 lists the optimization of preparation parameters, as well as the corresponding responses.

In view of the experimental conditions, the optimal combination of preparation parameters was chosen as follows: *BFC* is 0.34%, *BFL* is 6 mm, *AAR* is 6.57%. Three replicate samples were prepared and tested by the Marshall test method. The experimental results are summarized in Table 9. According to the relative error results, a very good agreement between the prediction and experiment can be observed, and the relative errors are less than 2%. This reveals that the design optimization of the preparation parameters of the modified asphalt mixture by RSM possesses favorable accuracy for Marshall test indices.

### 3.3. Comparative Analysis of Pavement Performance

**Figure 16:**
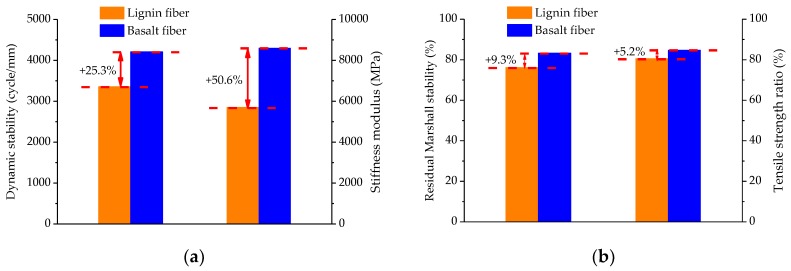
Pavement performance test results: (**a**) High-temperature dynamic stability and low-temperature cracking resistance; (**b**) Moisture stability.

In order to evaluate the pavement performance of optimal basalt fiber-modified asphalt mixture by RSM, wheel tracking, indirect tensile stiffness modulus, immersion Marshall and freeze-thaw splitting tests were conducted, and these pavement performance results can be used for comparison with the previous work [26]. Lignin fiber is a commonly used fiber in road engineering. In the previous work, SBS-modified asphalt mixture containing lignin fiber was prepared with a lignin fiber content of 0.4%, fiber length of 1.1 mm and asphalt-aggregate ratio of 6.8%. Then, the pavement performance of the lignin fiber-modified asphalt mixture was obtained. Figure 16 illustrates the pavement performance results, including dynamic stability, indirect tensile stiffness modulus, residual Marshall stability and tensile strength ratio.

As shown in Figure 16a, it can be observed from the comparative analysis that the dynamic stability of basalt fiber-modified asphalt mixture was improved by approximately 25.3%, and the indirect tensile stiffness modulus can be improved by up to 50.6% compared to lignin fiber-modified asphalt mixture. In general, a higher dynamic stability indicates a better high-temperature rutting resistance and a higher indirect tensile stiffness modulus is preferable for low-temperature cracking resistance. Therefore, this implies that the high-temperature rutting resistance and low-temperature cracking resistance can be greatly improved by using basalt fiber.

As illustrated in Figure 16b, the residual Marshall stability and tensile strength ratio of basalt fiber-modified asphalt mixture were improved by 9.3% and 5.2%, respectively. Higher residual Marshall stability and tensile strength ratio are usually desirable for better moisture stability. As a result, the moisture stability is improved by the addition of basalt fiber.

## 4. Conclusions

This study optimized the design of SBS-modified asphalt mixture reinforced with eco-friendly basalt fiber based on response surface methodology. The effects of the preparation parameters on Marshall test indices were discussed and analyzed. Meanwhile, the performance of basalt fiber-modified asphalt binder and mixture were also studied. The following conclusions can be drawn:

- A design optimization of basalt fiber and SBS-modified asphalt mixture is proposed based on response surface methodology—i.e., basalt fiber content: 0.34%, fiber length: 6 mm and asphalt-aggregate ratio: 6.57%—which possesses favorable and reliable accuracy compared with experimental results.
- Basalt fiber length has a more significant effect on Marshall stability than basalt fiber content and asphalt-aggregate ratio.
- Flow value presents a significant variation trend with asphalt-aggregate ratio and a larger flow value depends on a higher asphalt content.
- Basalt fiber content exhibits the most significant effect on air voids due to the lower specific gravity of basalt fiber. Additionally, voids in mineral aggregates also exhibit an approximately similar variation trend to air voids.
- Voids filled with asphalt are also more related to basalt fiber content; hence, a higher basalt fiber content should not be recommended in view of the interface adhesion.
- The spatial networking structure and absorption of light components in asphalt by basalt fiber can lead to better shear resistance and tensile properties, as well as lower temperature susceptibility of the asphalt binder. Meanwhile, the high-temperature stability, low-temperature cracking resistance and moisture stability can also be improved by the addition of basalt fiber.
- In comparing the cost of asphalt mixtures modified by basalt fiber and lignin fiber, the cost mainly depends on the fiber prices. The basalt fiber price is about 2~3 times that of the lignin fiber, while the performance of basalt fiber-modified asphalt mixture is better than that of lignin fiber-modified asphalt mixture. Therefore, in the long run, basalt fiber modified asphalt mixture would produce good economic benefits.

## Figures and Tables

**Table 1:** Basic physical properties of SBS-modified asphalt.

Properties	Measurement	Technical Criterion
Penetration @ 25 °C, 100 g, 5 s (0.1 mm)	57.9	40~60
Ductility @ 5 °C, 5 cm/min (cm)	24.9	≥20
Softening point (°C)	64.4	≥60
Flash point (°C)	286	≥230
Elastic recovery @ 25 °C (%)	88	≥75
Solubility (trichloroethylene, %)	100.2	≥99
RTFOT		
Mass loss (%)	0.22	±1.0
Penetration ratio @ 25 °C (%)	85	≥65
Ductility @ 5 °C (cm)	18.1	≥15

**Table 2:** Physical properties of aggregates and filler.

Index	Coarse Aggregate				Fine Aggregate	Filler
	13.2	9.5	4.75	2.36	2.36~0.075	<0.075
Crushed stone value (%)	22.45	23.13	23.62	24.16	−	−
Los Angeles abrasion loss (%)	26.47	25.99	25.78	26.14	−	−
Apparent specific gravity (g/cm^3^)	2.687	2.675	2.659	2.689	2.691	2.738
Water absorption (%)	0.99	1.13	1.57	1.76	−	−

**Table 3:** Physical properties of basalt fiber.

Index	Length	Diameter	Specific Gravity	Tensile Strength	Elongation at Break
Units	mm	µm	g/cm^3^	MPa	%
Value	3/6/9	13	2.55~2.65	≥3000	32

**Table 4:** Experimental design for face-centered central composite design (FCCD).

Factors		Units	Levels: Actual (Coded)		
			Low (−1)	Medium (0)	High (+1)
*X*_1_	*BFC*	%	0.25	0.40	0.55
*X*_2_	*BFL*	mm	3	6	9
*X*_3_	*AAR*	%	6.4	6.6	6.8

**Table 5:** Experimental design and test outcomes by face-centered central composite design (FCCD).

No.	Preparation Parameters			Responses				
	*BFC* *X*_1_ (%)	*BFL* *X*_2_ (mm)	*AAR* *X*_3_ (%)	*MS* *Y*_1_ (kN)	*FV* *Y*_2_ (mm)	*VA* *Y*_3_ (%)	*VMA* *Y*_4_ (%)	*VFA* *Y*_5_ (%)
1	0.40	6	6.6	9.98	3.052	4.69	18.35	74.45
2	0.25	3	6.4	7.48	3.053	2.80	16.79	83.32
3	0.55	3	6.8	6.23	3.328	7.19	20.47	64.88
4	0.55	6	6.6	9.92	2.778	5.99	19.46	69.22
5	0.40	6	6.8	9.33	4.153	5.89	19.36	69.58
6	0.40	6	6.6	10.56	3.054	4.78	18.42	74.05
7	0.55	9	6.8	7.19	3.053	7.55	20.78	63.67
8	0.25	6	6.6	10.21	3.190	2.75	16.69	83.52
9	0.40	3	6.6	8.72	3.191	4.37	18.08	75.83
10	0.40	6	6.6	10.48	3.053	4.69	18.35	74.44
11	0.40	6	6.6	10.29	3.051	4.82	18.46	73.89
12	0.55	3	6.4	6.63	2.641	6.05	19.57	69.09
13	0.25	9	6.8	7.82	4.291	4.28	17.97	76.18
14	0.25	9	6.4	8.41	2.916	3.13	17.07	81.66
15	0.25	3	6.8	6.89	4.428	3.96	17.70	77.63
16	0.40	6	6.6	10.38	3.053	4.65	18.32	74.62
17	0.40	6	6.4	9.61	2.916	5.07	18.73	72.93
18	0.40	9	6.6	9.04	2.915	5.02	18.63	73.05
19	0.55	9	6.4	8.11	2.503	6.37	19.85	67.91

**Table 6:** ANOVA results for quadratic models of modified asphalt mixture.

Responses		*R*-Squared	Adj. *R*-Squared	Adeq. Precision	*F*-Value	*p*-Value	Significant
*Y*_1_	*MS*	0.9834	0.9668	21.472	59.31	<0.0001	Yes
*Y*_2_	*FV*	0.9796	0.9591	25.279	47.94	<0.0001	Yes
*Y*_3_	*VA*	0.9966	0.9932	59.932	291.94	<0.0001	Yes
*Y*_4_	*VMA*	0.9968	0.9935	61.691	308.72	<0.0001	Yes
*Y*_5_	*VFA*	0.9953	0.9905	50.560	210.31	<0.0001	Yes

**Table 7:** ANOVA results for independent variables.

Responses	Factors	Sum of Squares	Degree of Freedom	Mean Square	*F*-Value	*p*-Value	Significant
*MS*	*BFC*	0.75	1	0.75	10.89	0.0092	**
	*BFL*	2.13	1	2.13	31.18	0.0003	***
	*AAR*	0.77	1	0.77	11.29	0.0084	**
	*BFC × BFL*	0.042	1	0.042	0.61	0.4533	–
	*BFC × AAR*	0.002	1	0.002	0.036	0.8541	–
	*BFL × AAR*	0.034	1	0.034	0.49	0.5000	–
	(*BFC*)^2^	0.48	1	0.48	7.02	0.0265	*
	(*BFL*)^2^	7.03	1	7.03	102.73	<0.0001	****
	(*AAR*)^2^	2.81	1	2.81	41.06	0.0001	***
*FV*	*BFC*	1.28	1	1.28	112.77	<0.0001	****
	*BFL*	0.093	1	0.093	8.18	0.0188	*
	*AAR*	2.73	1	2.73	240.79	<0.0001	****
	*BFC × BFL*	0.002	1	0.002	0.21	0.6553	–
	*BFC × AAR*	0.29	1	0.29	25.25	0.0007	***
	*BFL × AAR*	0.002	1	0.002	0.21	0.6599	–
	(*BFC*)^2^	0.053	1	0.053	4.66	0.0592	–
	(*BFL*)^2^	0.013	1	0.013	1.18	0.3054	–
	(*AAR*)^2^	0.46	1	46.27	40.82	0.0001	***
*VA*	*BFC*	26.34	1	26.34	2183.93	<0.0001	****
	*BFL*	0.39	1	0.39	32.50	0.0003	***
	*AAR*	2.97	1	2.97	246.26	<0.0001	****
	*BFC × BFL*	0.0001	1	0.0001	0.009	0.9252	–
	*BFC × AAR*	0.00001	1	0.00001	0.001	0.9750	–
	*BFL × AAR*	0.0001	1	0.0001	0.009	0.9252	–
	(*BFC*)^2^	0.30	1	0.30	24.49	0.0008	***
	(*BFL*)^2^	0.00003	1	0.00003	0.003	0.9555	–
	(*AAR*)^2^	1.67	1	1.67	138.25	<0.0001	****
*VMA*	*BFC*	19.35	1	19.35	2337.08	<0.0001	****
	*BFL*	0.29	1	0.29	34.50	0.0002	***
	*AAR*	1.82	1	1.82	220.23	<0.0001	****
	*BFC × BFL*	0.0002	1	0.0002	0.024	0.8799	–
	*BFC × AAR*	0.00005	1	0.00005	0.006	0.9398	–
	*BFL × AAR*	0.00005	1	0.00005	0.006	0.9398	–
	(*BFC*)^2^	0.22	1	0.22	26.41	0.0006	***
	(*BFL*)^2^	0.00002	1	0.00002	0.003	0.9593	–
	(*AAR*)^2^	1.29	1	1.29	155.82	<0.0001	****
*VFA*	*BFC*	456.17	1	456.17	1572.14	<0.0001	****
	*BFL*	6.86	1	6.86	23.63	0.0009	***
	*AAR*	52.76	1	52.76	181.84	<0.0001	****
	*BFC × BFL*	0.065	1	0.065	0.22	0.6478	–
	*BFC × AAR*	0.92	1	0.92	3.19	0.1079	–
	*BFL × AAR*	0.004	1	0.004	0.014	0.9085	–
	(*BFC*)^2^	10.03	1	10.03	34.56	0.0002	***
	(*BFL*)^2^	0.0005	1	0.0005	0.002	0.9661	–
	(*AAR*)^2^	27.97	1	27.97	96.38	<0.0001	****

Note: “****” *p* < 0.0001; “***” 0.0001 ≤ *p* < 0.001; “**” 0.001 ≤ *p* < 0.01; “*” 0.01 ≤ *p* < 0.05; “–” *p* ≥ 0.05.

**Table 8:** Target values of response variables.

Response	*MS* (*Y*_1_)	*FV* (*Y*_2_)	*VA* (*Y*_3_)	*VMA* (*Y*_4_)	*VFA* (*Y*_5_)
Units	kN	mm	%	%	%
Target value	Maximize	2~5	3~4	≥17	75~85

**Table 9:** Optimal preparation parameters and prediction vs. experiment.

Response		*BFC* *X*_1_ (%)	*BFL* *X*_2_ (mm)	*AAR* *X*_3_ (%)	*MS* *Y*_1_ (kN)	*FV* *Y*_2_ (mm)	*VA* *Y*_3_ (%)	*VMA* *Y*_4_ (%)	*VFA* *Y*_5_ (%)
Prediction		0.34	6.41	6.57	10.49	3.113	4	17.76	77.43
Experiment	1	0.34	6	6.57	10.35	3.118	3.99	17.66	77.41
	2	0.34	6	6.57	10.33	3.117	4.01	17.71	77.36
	3	0.34	6	6.57	10.39	3.113	4	17.67	77.36
	Mean	0.34	6	6.57	10.36	3.116	4	17.68	77.38
Relative error (%)		−	−	−	−1.24	0.10	0	−0.45	−0.06
